# Sclerokeratitis and Secondary Glaucoma in Relapsing Polychondritis in a 30-Year-Old Asian Male Patient: A Case Report

**DOI:** 10.7759/cureus.79128

**Published:** 2025-02-16

**Authors:** Lovely Keziah C Flores, Richmond R Siazon

**Affiliations:** 1 Ophthalmology, Ilocos Training and Regional Medical Center, San Fernando, PHL

**Keywords:** case report, relapsing polychondritis, scleromalacia perforans, secondary glaucoma, sensorineural deafness

## Abstract

Relapsing polychondritis (RPC) is an immune-mediated systemic disease characterized by recurring inflammatory episodes in cartilage structures, mainly in the ears, nose, and respiratory tract. It is associated with various systemic and ocular manifestations, such as scleritis, the most common ocular feature of RPC. Scleromalacia perforans (SP) is a rare and severe form of anterior scleritis that appears as a dark bluish discoloration and bulging of a thinned-out sclera in the absence of pain or redness. This case report describes a 30-year-old Asian male who presented a seven-year history of blurred vision and recurrent eye redness. Visual acuity (VA) was 15/200 and 20/63+2 on OD and OS, respectively. An eye exam showed diffusely hazy cornea, thinned-out sclera, and elevated intraocular pressure (IOP) in both eyes. A systems review revealed bilateral auriculitis and mixed hearing loss. He was clinically diagnosed with RP and started on systemic corticosteroids at 1 mg/kg/day. On follow-up, the patient showed resolution of conjunctival hyperemia; however, IOP remains elevated despite maximal therapy. Ocular manifestations in RPC are protean and may be the presenting symptoms of RPC, resulting in the possibility of delayed or missed diagnosis in the absence of chondritis. SP, corneal scarring, and secondary glaucoma are late sequelae and vision-threatening conditions that may pose significant therapeutic challenges to ophthalmologists. Multispecialty collaboration is warranted in the management of systemic complications of RPC.

## Introduction

Relapsing polychondritis (RPC) is an autoimmune disease of unknown etiology affecting the cartilage and proteoglycan-rich structures, such as the eyes and blood vessels. It is an immune-mediated multisystemic disease characterized by repeated inflammatory episodes in cartilage structures, mainly in the ears, nose, and respiratory tract, and is associated with various systemic manifestations. It is estimated that the incidence of diagnosed RPC is between 4.5 and 20 per million adults. It commonly affects middle-aged people (usually between 40 and 55 years old) but can occur at any age with a female predominance [1]. It is a rare disease usually seen in Caucasians but can affect all races [2]. Bilateral auricular chondritis is present in approximately 90% of cases and affects the ear cartilage with sparing of the lobes, resulting in deformity of the pinna resembling the “cauliflower ear” of professional boxers [2]. In addition, hearing loss, either conductive or sensorineural, is observed in up to 46% of the patients [1]. Other manifestations include saddle nose deformity, dysphonia due to laryngeal involvement, airway collapse, non-erosive inflammatory arthritis, and vasculitis. Aside from systemic effects, ocular manifestations most commonly occur as scleritis in approximately 20-60%, usually bilaterally. Other ocular findings may include episcleritis, uveitis, and keratitis [1,2]. Although the diagnosis is clinical, radiographic and histopathological confirmation may be necessary in patients presenting with atypical symptoms [3].

Scleritis, an inflammation of the outer white coat of the eye, is one of the most severe ocular manifestations of autoimmune diseases such as RPC. Scleromalacia perforans (SP) is a rare and severe form of anterior scleritis that presents as a blackish-blue hue through the thin visible sclera and is a late consequence in the course of the disease [4]. Other late-onset sequelae, including corneal blindness and secondary glaucoma, may develop in these patients, making its management particularly challenging because of the concomitant inflammation. In the Philippines, published case reports on RPC have presented auricular and respiratory problems, but none have described ocular complications [5,6]. This rare case report describes the case of a young adult male of Asian descent, who initially presented with recurrent visual symptoms and later developed serious potentially blinding complications. This report aims to raise awareness among ophthalmologists by highlighting the recurrent nature of ocular symptoms in RPC that are visually destructive if not recognized early, to avoid misdiagnosis that can lead to delays in management, and to emphasize the importance of multispecialty collaboration in the long-term management of potential systemic complications.

## Case presentation

**Figure 1:**
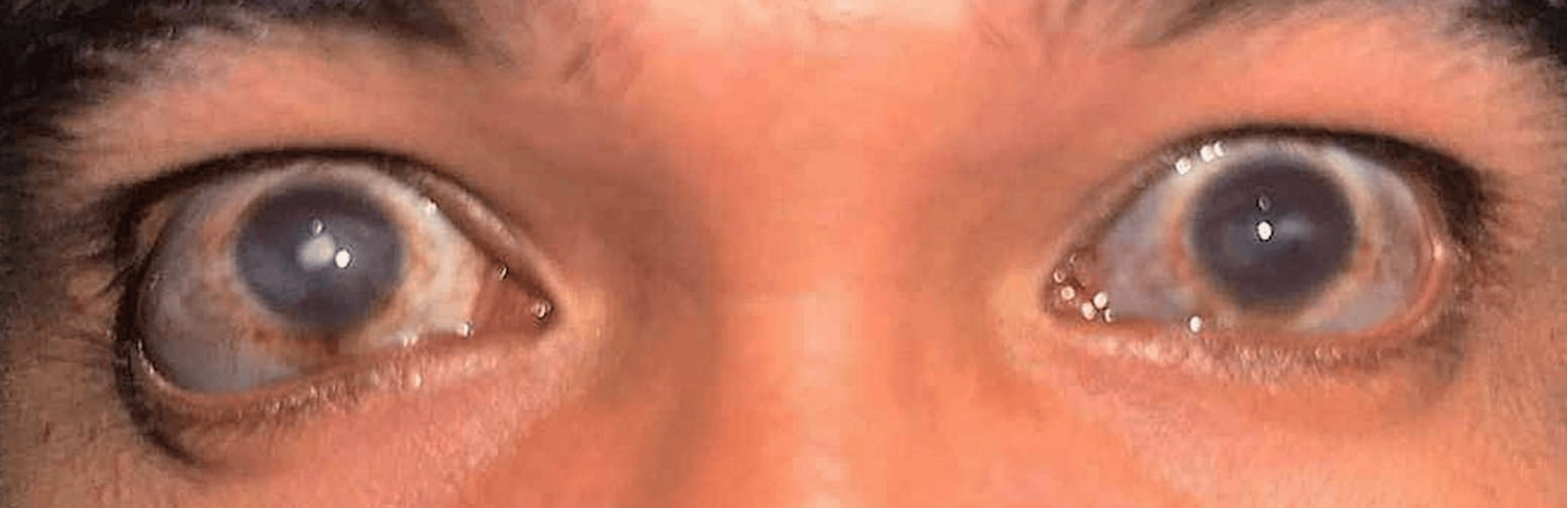
A 30-year-old male with bulging and darkening of sclera and corneal scarring in both eyes. Both eyes are in the primary position of gaze, showing a dark bluish discoloration of the anterior 360-degree sclera, with areas of focal protrusion most pronounced in the inferotemporal areas of both eyes, worse in the right eye. A dense central corneal scar can also be seen on both eyes but worse in the right eye.

**Figure 2:**
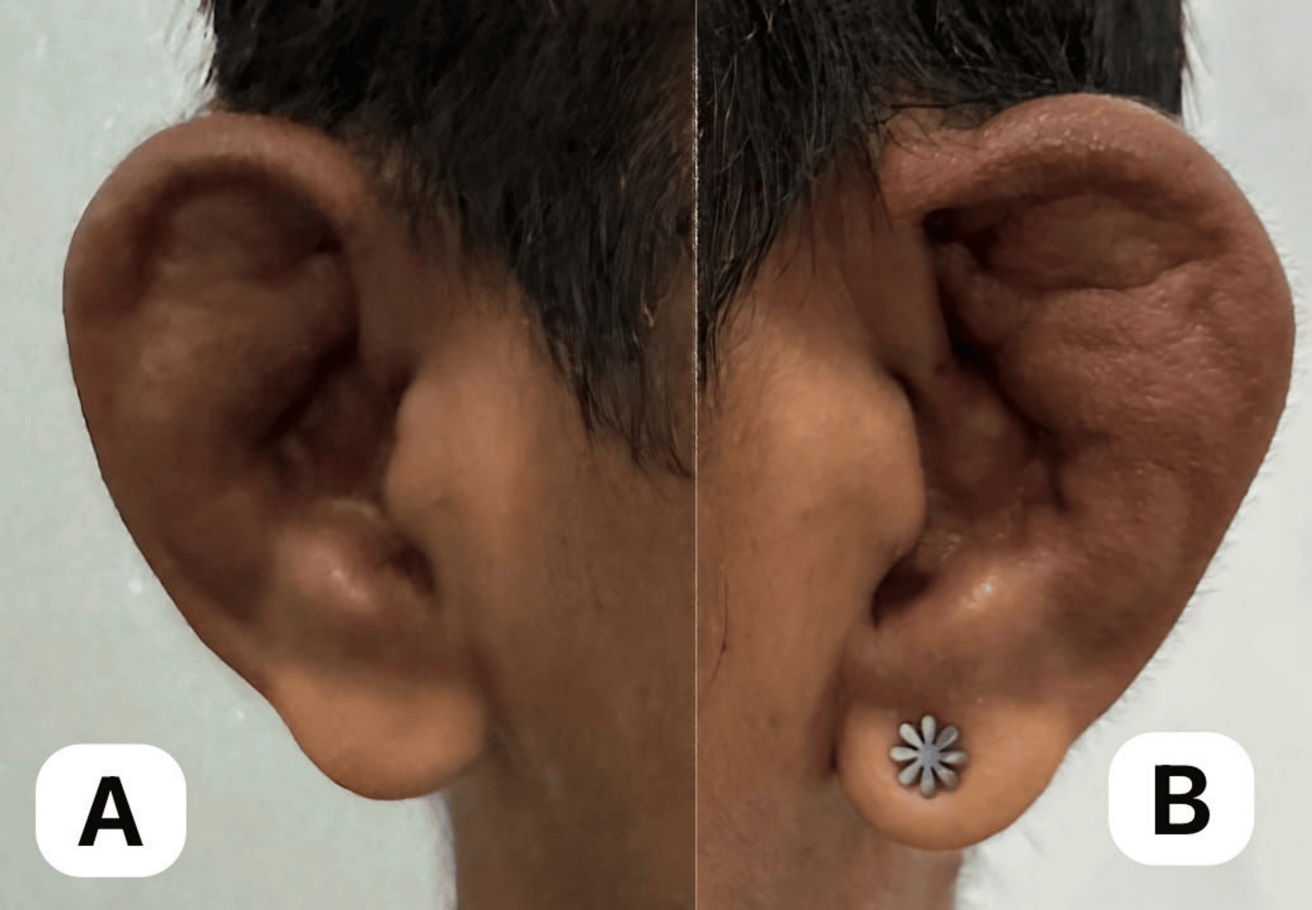
Right ear (A) and left ear (B), both showing auricular chondritis. Both ears show roughness of the skin and dark discoloration and swelling of the pinna with sparing of the lobes.

A 30-year-old Asian male presented with a seven-year history of recurrent redness and blurred vision in both eyes, which was initially treated at a private clinic with topical antibiotics + steroids that provided temporary relief. In many instances, the recurrences necessitated repeat outpatient visits. On presentation, there was progressive darkening and bulging of the sclera in both eyes, prompting consultation with our outpatient department (Figure 1). There was no accompanying eye pain, photophobia, or foreign body sensation. A systems review revealed bilateral ear deformities with skin discoloration and mixed hearing loss (Figure 2, A-B). He denied joint pain, deformity, and difficulty in breathing, or hoarseness. The patient had no history of ocular trauma or surgery. Past medical, family, and drug histories were negative.

**Figure 3:**
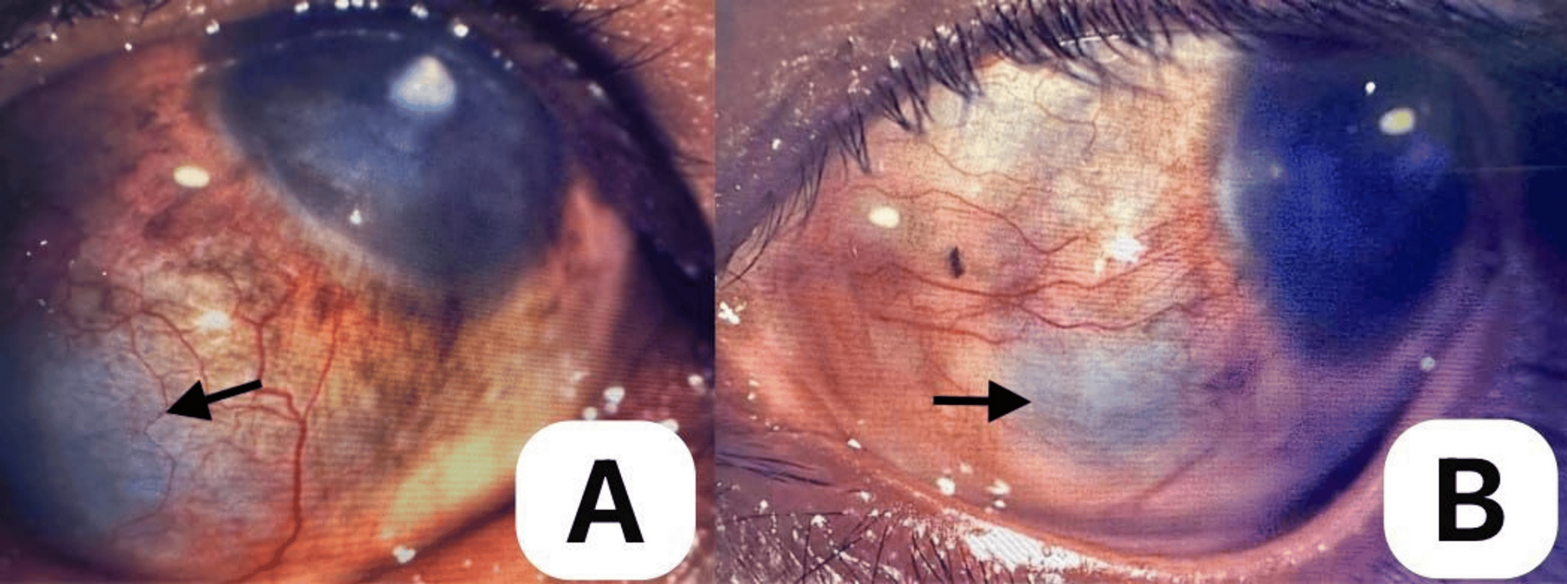
Right eye (A) and left eye (B), showing dark bluish hue of thinned-out sclera (black arrows). Right eye (A) looking superonasally, revealing a thinned-out sclera and an area of protrusion inferotemporally, surrounded by prominent episcleral and deep scleral vessels; and left eye (B) on left gaze, showing thinned-out sclera inferonasally with surrounding prominent vessels.

**Figure 4:**
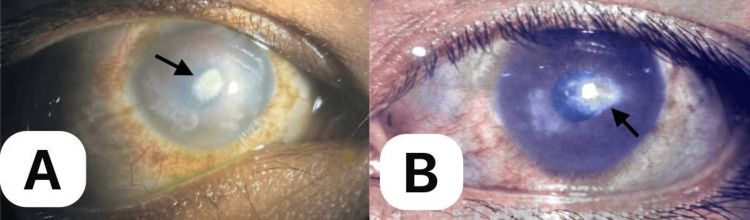
Right (A) and left eye (B), showing a diffusely hazy cornea with denser central corneal scarring (black arrows). The right eye (A) in slight left gaze shows a diffusely hazy cornea with a dense central corneal scar (black arrow) precluding view of the posterior pole. The left eye (B) in primary gaze shows focal areas of scarring, most pronounced in the central cornea (black arrow). Anterior chamber structures are visible in the left eye.

Ophthalmological examination revealed that the visual acuity (VA) of the right eye (OD) was 15/200 and the left eye (OS) was 20/63+2, which did not improve in pinhole. Intraocular pressure (IOP) was 20 and 37 mmHg for OD and OS, respectively. The pupils were 2-3 mm reactive to light with no relative afferent pupillary defect (RAPD). Slit lamp examination revealed a dark bluish hue through the thin anterior 360° sclera, with focal areas of protrusion in the oculus uterque (OU), most pronounced in the inferolateral area (Figure 3, A-B). Prominent episcleral and deep scleral vessels and mild conjunctival hyperemia were also noted in both eyes. Both corneas were diffusely hazy, becoming opaque centrally precluding the view of the posterior pole in the OD (Figure 4, A-B). No fluorescein dye uptake was observed. The anterior chamber was quiet with no cells, flares, posterior synechiae, or lens opacity in the OU. A gonioscopy revealed open angles for both eyes. The cup-to-disc ratio was within normal limits in the left eye. The funduscopic findings for OS were unremarkable. B-scan ultrasonography of OD was normal.

**Figure 5:**
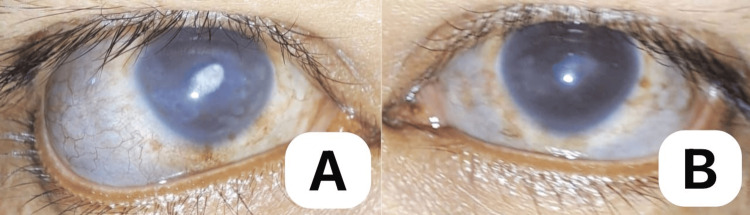
Right (A) and left (B) eyes showing resolution of conjunctival hyperemia after a month of oral steroid treatment. The right eye (A) and left eye (B) in primary gaze, showing resolution of conjunctival hyperemia and less prominent episcleral and deep scleral vessels. Areas of thinned-out sclera and corneal scars remain the same in both eyes.

**Figure 6:**
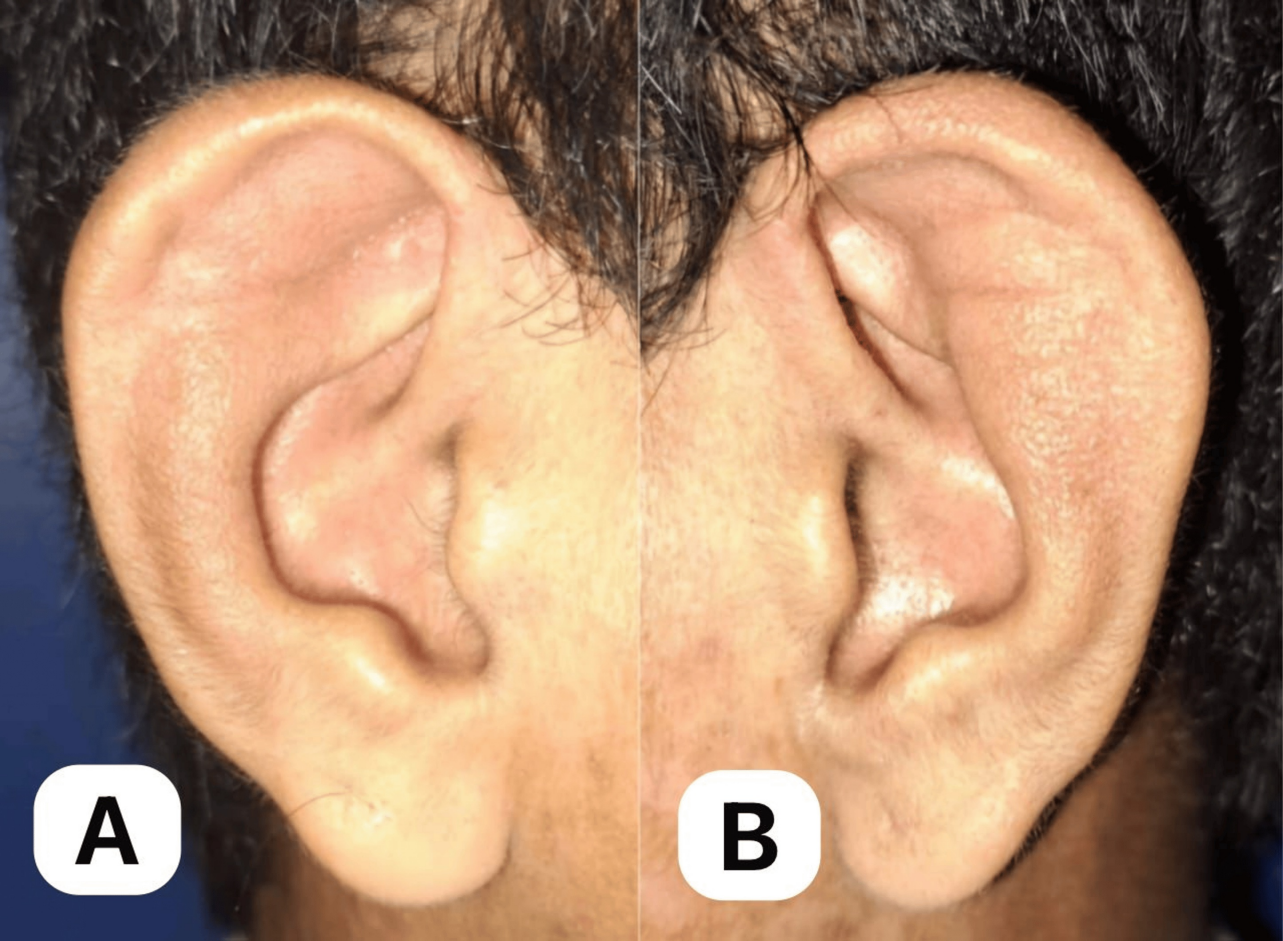
The right (A) and left (B) ears showing resolution of auricular chondritis. The right (A) and left (B) ears show the disappearance of dark skin discoloration and the resolution of inflammation of the pinna.

A working impression of sclerokeratitis probably autoimmune in origin was made. Autoimmune and infectious workups were also performed. Complete blood count (CBC), urinalysis, and fecal analysis revealed leukocytosis. Chest radiography results were negative. The C-reactive protein (CRP) level and erythrocyte sedimentation rate (ESR) were markedly elevated levels at 58.17 and 53, respectively. Antinuclear antigen (ANA), rheumatoid factor (RF), HbsAg, and anti-HbC test results were negative (Table 1). Venereal disease research laboratory (VDRL) and Mantoux tests were not performed because of financial constraints. The patient was referred to the Rheumatology Department. A referral to the otorhinolaryngology service confirmed the presence of bilateral auricular chondritis of the ear cartilage and mixed hearing loss. He was advised to undergo a tissue biopsy of the pinna but was refused by the patient. He was initially treated with topical tobramycin/dexamethasone eye drops every four hours and topical lubricants quater in die (QID) on both eyes. Brimonidine + timolol eyedrops were administered to control high IOP. Oral prednisone (1 mg/kg/day) was administered after ruling out an infectious etiology. On follow-up, the patient showed clinical improvement with less conjunctival hyperemia; however, the best-corrected VA remained the same (OD 15/200, OS 20/63+2). There were no signs of impending scleral rupture or active inflammation. The patient was maintained on the current topical regimen for glaucoma and lubricants and oral steroids were tapered off and started on methotrexate 2.5 mg/tablet QID. He was advised protective eyewear at all times. Subsequent follow-up after several months revealed a resolution of inflammation of the eyes and ears (Figure 5 A-B, Figure 6 A-B). The patient returned to his daily activities while being maintained on a topical antiglaucoma medication and oral methotrexate 10 mg/week. He is being co-managed by the Rheumatology and Ophthalmology under the External Disease and Cornea and Glaucoma service, which involves a possible implant of a glaucoma draining device shortly to address the high IOP.

**Table 1:** Laboratory test results. HBsAg (hepatitis B surface antigen); Anti-HCV (hepatitis C antibody); CRP (C-reative protein); ESR (erythrocyte sedimentation rate); RF (rheumatoid factor); ANA (antinuclear antibodies); Typidot IgM, IgG (typhoid IgM, IgM)

Laboratory test	Result	Normal values
HBsAg	Nonreactive	Nonreactive
Anti-HCV	Nonreactive	Nonreactive
CRP	58.17 mg/L (markedly elevated indicating inflammation)	10 mg/L
ESR	53 mm/hr (markedly elevated indicating inflammation)	0-15 mm/hr
RF	Negative	Negative
ANA	Negative	Negative
Creatinine	116.67 (slightly elevated)	53-106 mmol/L
Typhidot IgM, IgG	Nonreactive	Nonreactive

## Discussion

The clinical features of our patient, such as auricular deformity, sensorineural and conductive hearing loss, and scleromalacia perforans, were consistent with the diagnosis of RPC. According to Michet et al., patients with suspected RPC must have inflammation in at least two cartilage locations or one chondritis site with at least two of the following features: ocular inflammation, seronegative arthritis, hearing loss, and vestibular dysfunction [7]. There are no specific diagnostic tests to confirm the diagnosis; however, laboratory tests, such as CBC, ESR, CRP, autoimmune and infectious screening, including RF, ANA, anticytoplasmic antibody (c-ANCA), and VDRL test, and urinalysis are commonly requested to rule out concomitant or associated autoimmune diseases [1]. In our patient, inflammatory markers were markedly elevated, which is typical of autoimmune diseases. Imaging studies such as computed tomography (CT), magnetic resonance imaging (MRI), and color Doppler ultrasonography, have been described as useful tools for estimating local inflammation and diagnosing RPC. Chest CTs and MRIs can show thickening and narrowing of the airways and cartilage destruction early in the disease process, leading to prompt diagnosis and aid in the evaluation of therapeutic response [5,6]. Functional airway abnormalities such as air trapping can also be detected by CT scans or pulmonary function tests (PFT), which can aid in early disease recognition [2]. However, imaging studies of the laryngotracheal structures were not performed in this case due to the absence of respiratory symptoms and normal laryngoscopic examination.

Although rheumatoid arthritis (RA) is the most common autoimmune cause of scleritis, bilateral auricular chondritis, absence of joint involvement, and RF seronegativity make RA an unlikely etiological cause [8]. Granulomatosis with polyangiitis (GPA) a life-threatening vasculitis presenting with eye manifestations was also ruled out. After RA and GPA, RPC is the third most common systemic association, occurring in 6.4% of patients with autoimmune scleritis, and is considered in the differential diagnoses [9].

The pathogenesis of RPC has not been fully explained, although the autoimmune response to type II collagen has been implicated in both humoral and cellular immunity involved in cartilage destruction [4,9]. The exact trigger factor remains unknown; however, it was reported that trauma to the pinna of the ear may herald RPC in genetically predisposed individuals [2]. T helper cells and cytokines play crucial roles in the cascade of events that lead to the autoimmune-mediated inflammatory destruction of cartilage and proteoglycan-rich tissues. Treatment aims to interrupt specific steps in this cascade [4].

The histopathology of chondritis, which is the main inflammatory finding in RPC, may reveal the absence of cartilage, chondrocyte apoptosis, fibrosis, and focal calcification [1,2,9]. Proteoglycan-rich structures, such as the sclera and cornea, are often involved and come into attack by the inflammatory cells, resulting in keratitis, scleritis, episcleritis, and uveitis. Ocular manifestations can be the presenting manifestation of RPC in 21% of cases, as in our case, preceding cartilage inflammation [10]. While most autoimmune diseases have female preponderance, ocular symptoms in RPC are seen more frequently and more severely in the male population [1,3]. The exact reason for this is unclear [10]. Episcleritis and scleritis are the two most frequent ocular manifestations of RPC, occurring in almost half of the patients [11]. Early on, ocular symptoms may be non-specific and may not arouse suspicion despite the recurrent nature of the presenting symptoms. In addition, the long interval and unpredictability in the appearance of other symptoms, such as nasal and auricular chondritis, arthritis, respiratory distress, and vestibular disorders may result in diagnostic delays or misdiagnosis of RPC [6].

We surmised that the patient’s long history of recurrent eye redness accompanied by blurred vision might have been a manifestation of sclerokeratitis, resulting in damage to the sclera (scleromalacia perforans) and cornea (corneal scarring). Scleritis is a frequent cause of visual morbidity owing to its potentially sight-threatening complications such as cataracts, glaucoma, and retinal and choroidal problems [8,12]. The dense central corneal scar observed in this patient may be an old non-healing ulcer that rarely occurs in RPC, as observed in only three previously reported cases [10]. Secondary glaucoma is often due to angle closure secondary to rotation of the ciliary body, as well as ciliary body edema [4,13]. However, in our case, glaucoma was attributable to alterations in scleral architecture due to scleritis, causing an elevated intraocular pressure that was unresponsive to medical therapy. Due to the rarity of RPC, there are no established treatment guidelines yet [2,4]. However, the goals of treatment are to control the symptoms, prevent recurrences and complications, and preserve vision and hearing to maintain a good quality of life for these patients [1]. Corticosteroids are the mainstay treatment for this condition. Immunosuppressants, such as methotrexate and azathioprine, are also used as maintenance treatments in combination with low-dose corticosteroids [1,5,12]. Methotrexate offers the advantage of ready availability and lower cost. Biologic agents including tumor necrosis factor (TNF) inhibitors showed an overall response rate of 63% in one study, but they were used in combination with corticosteroids [14]. Biologic medications are expensive and difficult to produce limiting their availability. For our patient, low-dose methotrexate (10 mg/week) was sufficient to control the symptoms, and monitoring is being done by the rheumatology service. Surgical options such as corneal transplant and placement of glaucoma drainage device (GDD) are limited and not readily available in most centers and are associated with a high risk of failure owing to the inflammatory nature of the disease. The prognosis is good for mild disease as in this case, but long-term follow-up with an ophthalmologist and otorhinolaryngologist is recommended to monitor and manage eye and ear complications. Severe disease has potentially fatal complications, especially respiratory compromise. Up to 50% of patients eventually develop tracheobronchial tree collapse from chondritis of the respiratory tract causing obstructive respiratory failure, the most common cause of mortality [2,6,14]. Early recognition and timely management by a multispecialty team of ophthalmologists, rheumatologists, pulmonologists, and cardiologists is therefore warranted.

## Conclusions

RPC is a rare, autoimmune, and inflammatory disease of the cartilaginous and proteoglycan-rich tissues of the body. It is an important cause of eye disease characterized by recurrences and complications that threaten sight, such as corneal blindness, scleromalacia perforans, and secondary glaucoma. RPC poses diagnostic challenges for ophthalmologists, especially if presenting ocular manifestations are nonspecific and in the absence of chondritis, which is the hallmark of this condition. Surgical options are limited and challenging due to the inflammatory nature of the disease. A high index of suspicion is paramount for early recognition of systemic signs and timely intervention to prevent complications. A multidisciplinary approach should be adopted in collaboration with a team of otorhinolaryngologists, rheumatologists, cardiologists, and immunologists to manage potential systemic complications.
